# Endothelial Dysfunction in Diabetes

**DOI:** 10.3390/biomedicines8070182

**Published:** 2020-06-29

**Authors:** Yusuke Takeda, Keiichiro Matoba, Kensuke Sekiguchi, Yosuke Nagai, Tamotsu Yokota, Kazunori Utsunomiya, Rimei Nishimura

**Affiliations:** 1 Division of Diabetes, Metabolism, and Endocrinology, Department of Internal Medicine, The Jikei University School of Medicine, Tokyo 105-8461, Japan; ms05-takeda@jikei.ac.jp (Y.T.); k.sekiguchi.0322@gmail.com (K.S.); y.nagai@jikei.ac.jp (Y.N.); yokotat@jikei.ac.jp (T.Y.); rimei@jikei.ac.jp (R.N.); 2 Center for Preventive Medicine, The Jikei University School of Medicine, Tokyo 105-8461, Japan; kazu-utsunomiya@jikei.ac.jp

**Keywords:** endothelial dysfunction, insulin resistance, macrophage polarity

## Abstract

Diabetes is a worldwide health issue closely associated with cardiovascular events. Given the pandemic of obesity, the identification of the basic underpinnings of vascular disease is strongly needed. Emerging evidence has suggested that endothelial dysfunction is a critical step in the progression of atherosclerosis. However, how diabetes affects the endothelium is poorly understood. Experimental and clinical studies have illuminated the tight link between insulin resistance and endothelial dysfunction. In addition, macrophage polarization from M2 towards M1 contributes to the process of endothelial damage. The possibility that novel classes of anti-hyperglycemic agents exert beneficial effects on the endothelial function and macrophage polarization has been raised. In this review, we discuss the current status of knowledge regarding the pathological significance of insulin signaling in endothelium. Finally, we summarize recent therapeutic strategies against endothelial dysfunction with an emphasis on macrophage polarity.

## 1. Introduction

Diabetes is a global health problem, characterized by defective insulin secretion and resistance to insulin. According to the International Diabetes Federation (IDF), the number of people with diabetes is estimated to rise from 425 million at present to more than 600 million by 2045 [1]. Diabetes carries a significant risk of microvascular pathologies, such as retinopathy, nephropathy, neuropathy, and atherosclerotic diseases. Indeed, the relative risk of cardiovascular disease increases by two- to four-fold in patients with diabetes compared to non-diabetes patients [2]. Endothelial dysfunction is an early marker for atherosclerosis, preceding angiographic or ultrasonic evidence of atherosclerotic plaque [3,4]. In addition, accumulating evidence implicates endothelial dysfunction as an event seen even in patients with prediabetic conditions, such as impaired fasting glucose (IFG) and impaired glucose tolerance (IGT) [5].

The vascular endothelium functions as a structural barrier between the lumen and vessel wall. Studies over the past decade have also shown that the endothelium secretes numerous growth factors and cytokines that regulate multiple vascular functions (e.g., vascular tone, proliferation of vascular smooth muscle, platelet aggregation, coagulation, and fibrinolysis). Furthermore, the endothelium mediates vasoconstriction by secreting mediators, such as endothelin-1 and thromboxane A2. In contrast, substances such as nitric oxide (NO), prostacyclin, and endothelium-derived hyperpolarizing factor (EDHF) regulate vasodilation [2]. NO, the primary contributor synthesized from L-arginine by endothelial NO synthase (eNOS), regulates the endothelium-dependent relaxation of arteries [6].

Endothelial dysfunction is characterized by a loss of molecular functions in endothelial cells. Factors promoting this event include metabolic disorders (e.g., diabetes [7,8], obesity [9], dyslipidemia [10]), smoking [11], a high salt intake [12], lack of exercise [13], and menopause [14]. The release of reactive oxygen species (ROS) and the generation of oxidative stress are considered critical factors for the pathogenesis of diabetic vascular complications. While endothelial dysfunction is associated with various pathological aspects, including local inflammation [15,16] and oxidative stress [17,18], the pivotal mechanisms are the decrease of NO production and inactivation of NO [19]. The inactivation of NO results from oxidative stress caused by uncoupling of eNOS [20] and an increase in ROS-generating enzymes, including nicotinamide adenine dinucleotide phosphate-oxidase (NADPH) oxidase (NOX), cyclooxygenases (COX), and xanthine oxidase (XO) [21,22,23].

A variety of clinical methods for assessing the endothelial function are used. Previous studies to assess the endothelial function in humans have often evaluated NO-dependent vasodilation. Measuring the changes in the diameter and blood flow of the coronary artery in response to intra-coronary infusion of acetylcholine is considered the standard method [24]. Non-invasive methods for measuring the endothelial function have been evaluated in previous studies [25]. One of the most commonly applied techniques is flow-mediated dilation (FMD), which is evaluated by brachial artery ultrasound [26]. This method is well-trusted and relevant to cardiovascular risk factors [27] but is highly dependent on the experience level of the operators, who need special training [28]. The analysis of the pulse amplitude tonometry (PAT) in the index finger after reactive hyperemia has been considered as another non-invasive method for assessing the endothelial function [29]. Elevation of plasma concentrations of biomarkers of hemostasis, inflammation, and oxidative stress are also used as indices suggesting endothelial dysfunction [24]. Circulating levels of markers such as P- and E-selectin, ICAM-1, VCAM-1, plasminogen activator inhibitor-1 (PAI-1), oxidized low-density lipoprotein (oxLDL), and asymmetrical dimethylarginine (ADMA) have been used as markers of endothelial dysfunction [24].

We herein review the underlying mechanisms of endothelial dysfunction in diabetes and discuss how endothelial metabolism is targeted by the clinical agents.

## 2. Insulin Resistance and Endothelial Dysfunction

Insulin plays a vital role in the maintenance of vascular homeostasis. Insulin resistance is defined as an impaired biologic sensitivity and/or responsiveness to insulin stimulation in target tissues including the muscle, adipose tissue, and liver. Substantial evidence supports insulin resistance as the essential pathophysiologic impairment responsible for metabolic and cardiovascular disorders, collectively known as metabolic syndrome. Disturbance of insulin signaling eventually leads to glucose intolerance, diabetes, dyslipidemia, and coronary artery disease. Over the past two decades, many studies have focused on mechanisms provoking endothelial dysfunction, including ROS-mediated eNOS uncoupling, loss of NO bioavailability, and hyperglycemia-induced apoptosis of vascular endothelium, which ultimately leads to impaired vascular relaxation, a common biomarker of endothelial dysfunction. Understanding the endothelial control of metabolism in detail may aid in the development of novel approaches for intervention in obesity and obesity-related diseases.

### 2.1. Insulin Signaling in Endothelium

Insulin binds to the cell surface receptor known as the insulin receptor (IR). Activated IR phosphorylates intracellular substrates, such as insulin receptor substrate (IRS) family members, Shc proteins, and Gap-1 [30]. In humans, three isoforms of IRS—1, 2, and 4—have been shown to play important roles that vary depending on the cell type and metabolic conditions. For example, IRS-1 regulates insulin action in skeletal muscle as evidenced by findings that genetic ablation of IRS-1 results in insulin resistance and hypertriglyceridemia. IRS-2 functions as a regulator of insulin action in liver and pancreatic β cells. Intriguingly, IRS-2-deficient mice are more susceptible to diabetes than IRS-1 knockout mice because of the impairment of insulin secretion [31], indicating that IRS-2 contributes to the molecular basis for diabetes. The phosphorylated IRS tyrosine activates phosphoinositide-3 kinase (PI3-K) and then converts phosphatidylinositol (3,4)-bisphosphate (PIP2) to phosphatidylinositol (3,4,5)-trisphosphate (PIP3). PIP3 initiates a cascade of serine kinases, resulting in the recruitment of phosphoinositide-dependent kinase-1 (PDK-1) and Akt to the membrane, where they are activated [32]. Activation of Akt greatly influences cellular functions by regulating NO production, angiogenesis, and glucose metabolism [33].

Both IRS-1 and -2 are expressed in the endothelium [34]. Akt activation promotes the cell survival and proliferation of tumor vasculature [35]. Under pathophysiological conditions including obesity and insulin resistance, selective endothelial insulin resistance is promoted by proteasomal degradation of IRS-2 [34]. In the setting of insulin resistance, the reduction of endothelial proliferation results in atherosclerosis, diminished collateral angiogenesis in occluded coronary arteries, and reduced reendothelialization [2]. Furthermore, emerging evidence has shown that the proangiogenic role of Akt is induced by the generation of hypoxia-inducible factor α (HIFα). HIFα activation leads to the expression and subsequent production of angiogenic factors, such as vascular endothelial growth factor (VEGF). Akt’s ability to enhance the rate of glycolysis is dependent on HIFα and the subsequent expression of glycolytic enzymes [36].

Another insulin signaling pathway proceeds from Shc, which causes activation of the small GTP binding protein Ras and then initiates a phosphorylation cascade involving mitogen-activated protein kinase (MAPK). The MAPK pathway is associated with endothelial cells, mediating the secretion of ET-1 [37]. Insulin signal pathways form an extremely complicated network and multiple feedback loops. In other words, while MAPK pathways are weakly associated with regulating metabolic functions, PI3-kinase-dependent pathways function as pivotal branches to mediate the metabolic actions of insulin.

### 2.2. Insulin Resistance in the Endothelium

Insulin resistance is characterized by the deficiency in metabolic actions of insulin. A disorder of the PI3-K/Akt pathway results in a lack of insulin sensitivity in peripheral tissues. The MAPK pathway is strongly activated with compensatory hyperinsulinemia to produce inflammatory mediators (i.e., ICAM-1, VCAM-1, and E-selectin) when the PI3-K/Akt axis is downregulated [38]. The imbalance between these two signals leads to endothelial dysfunction, characterized by a decreased production of NO and increased generation of ET-1 in endothelial cells [39,40].

NOX is a key molecule in the development of endothelial dysfunction and is a major source of ROS production in endothelial cells. Type 2 diabetes is characterized by impaired control of the redox environment with overproduction of ROS [41]. The main factors playing a protective role are eNOS and NO. The biological balance at the endothelium is maintained by vasodilatory substances (i.e., prostaglandins, NO) and vasoconstricting factors (i.e., ET-1, angiotensin II). The activated PI-3/Akt pathway induces the phosphorylation of eNOS, transformation of L-arginine to L-citrulline, and production of NO. NO exerts a vasoprotective role by inhibiting the proliferation of vascular smooth muscle cells, expression of inflammatory cytokines, and platelet aggregation. In contrast, the lack of NO generation leads to the enhanced production of inflammatory and thrombotic cytokines [42]. Taken together, these findings indicate that the involvement of endothelial dysfunction and insulin resistance in pathological disorders contributes to the impairment of the cellular glucose uptake, NO-dependent vasodilation, enrichment of oxidative stress, and inflammation.

Elevation of circulating cytokine levels is strongly associated with insulin resistance and contributes to endothelial dysfunction. Increased levels of cytokines, including C-reactive protein (CRP), TNF-α, and interleukin-6 (IL-6), inhibit insulin-stimulated NO production by decreasing the eNOS expression, leading to the inhibition of the PI3K/Akt/eNOS pathway [43,44]. Obesity and type 2 diabetes are associated with elevated levels of leptin and resistin, which induce increases in TNF-α and IL-6 [45]. In addition, leptin enhances the serine phosphorylation of IRS-1, thereby disturbing insulin signaling through the PI-3K/Akt pathway [46]. In contrast, resistin reduces the expression of eNOS [47]. Although adiponectin and ghrelin stimulate NO production through the PI-3K/Akt signaling pathways and enhance the NO bioavailability, both cytokines are known to be reduced in patients with obesity or type 2 diabetes [48,49].

## 3. Crosstalk between Macrophage Polarization and Endothelial Cells

Macrophages and endothelial cells are closely related to each other. Endothelial cells produce cytokines pivotal for the differentiation and growth of macrophages. Macrophages constitute an important line of defense against infection and are essential for tissue repairing as well as wound healing [50,51]. These broad actions are mediated through macrophage conversion induced by environmental signals, such as lower temperatures and the secretion of colony-stimulating factor 1 (CSF-1) and interleukin (IL)-4. There are two types of macrophages: the proinflammatory M1 phenotype (classic activation) and the anti-inflammatory M2 phenotype (alternative activation). Adipose tissue macrophages (ATMs) from obese mice and humans are polarized toward an M1 phenotype, with the upregulation of tumor-necrosis factor (TNF) and inducible NO synthase (iNOS). In contrast, “lean” ATMs express high levels of M2 genes, including IL-10, Ym1, and Arginase 1 [52]. Emerging evidence indicates that proinflammatory M1 polarization induces adipose inflammation [53,54]. Consistently, a lack of M1 macrophages improves insulin sensitivity in obese mice [55,56]. In contrast, deletion of M2 macrophages has been shown to contribute to insulin resistance in wild-type mice [57]. These findings imply that macrophage polarization is implicated in metabolic disturbance.

**Figure 1:**
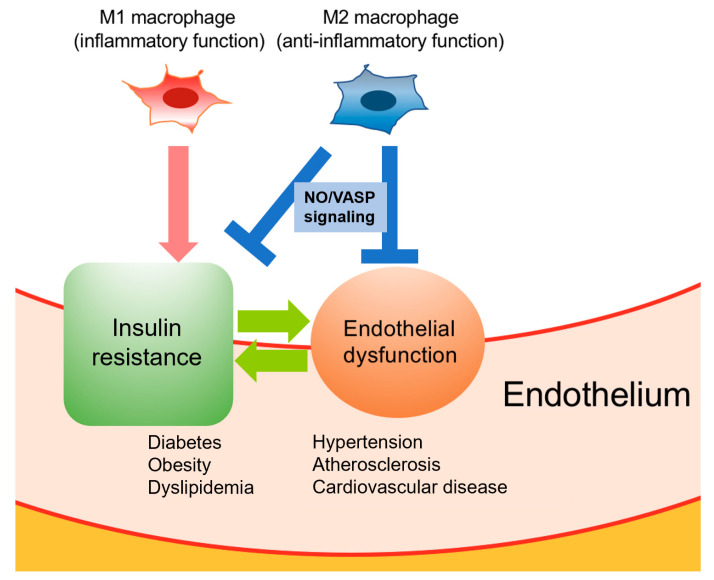
The metabolic network between macrophage polarization, insulin resistance, and endothelial cells. Macrophages play pleiotropic functions in the endothelium. Proinflammatory M1 macrophages are stimulated by LPS, IFN-γ, and TNF-α and promote the secretion of inflammatory cytokines, including IL-1, IL-6, and TNF-α. In contrast, anti-inflammatory M2 macrophages are stimulated by IL-4 and IL-10 and secrete anti-inflammatory cytokines, such as IL-10 and TGF-β. Through NO/VASP signaling, M2 macrophages contribute to the suppression of inflammation in the endothelium, leading to the improvement of insulin resistance and endothelial dysfunction. NO, Nitric oxide; VASP, Vasodilator-stimulated phosphoprotein.

NO exerts anti-inflammatory and antithrombotic effects. These actions are mediated by the activation of soluble guanylate cyclase, which in turn activates cyclic guanosine monophosphate (cGMP)-dependent protein kinase (PKG) through increased levels of cytoplasmic cGMP [58]. Vasodilator-stimulated phosphoprotein (VASP), a downstream target of PKG, has been identified as a regulator controlling cytoskeletal remodeling and cell migration [59]. Previous studies focusing on insulin resistance have revealed the endothelial NO/VASP-mediated suppression of inflammation in adipose tissue and liver [60,61]. Of note, activation of NO/VASP signaling promotes a phenotypic change into an M2 macrophage state. Conversely, a high-fat diet (HFD) attenuates M2 polarization and induces M1 activation in Kuppfer cells, which leads to insulin resistance in the liver [58]. Taken together, these findings suggest that a therapeutic approach targeting the NO/VASP pathway would promote anti-inflammatory actions and may thus be effective for managing metabolic disorders, including obesity and diabetes (Figure 1).

## 4. Targeting Endothelial Dysfunction

The ultimate goal of the treatment of diabetes is to prevent microvascular and macrovascular complications. The endothelium lining the inner wall of the vasculature modulates basic hemostatic functions, including the circulation of blood cells, vascular tone, platelet activity, and inflammation. Endothelial dysfunction is considered an early predictor of future cardiovascular events and atherosclerosis. Growing knowledge concerning the diverse functions of the endothelium has focused attention on therapeutic strategies that may improve the endothelial function. From a clinical standpoint, a large amount of experimental evidence supports the notion that therapies targeting endothelial dysfunction reduce cardiovascular mortality and morbidity. It is important to consider whether or not drugs used in the clinical management of type 2 diabetes exert positive and pleiotropic effects on the endothelium independent of the glucose-lowering action. While statins have been reported to exert vascular protective effects that are independent of lowering the LDL-cholesterol level, some anti-diabetic agents have recently been suggested to exert beneficial effects against endothelial dysfunction. In addition to traditional drugs, clinical and experimental data support the possibility that novel classes of anti-hyperglycemic agents have beneficial effects on the endothelial function and macrophage polarization.

### 4.1. SGLT2 Inhibitors

SGLT2 inhibitors block the glucose uptake in the renal proximal tubule of the nephron, resulting in the induction of glycosuria and decreased blood glucose levels. Recent trials, such as the EMPA-REG-OUTCOME and the CANVAS Program have revealed that SGLT2 inhibitors, i.e., empagliflozin and canagliflozin, attenuate cardiovascular events and reduce the death rate compared to the patients treated with placebo [62,63]. SGLT2 inhibitors have been suggested to exert beneficial actions on the endothelial function. For example, Shigiyama et al. clearly demonstrated that dapagliflozin add-on therapy on metformin improved the endothelial function by improving the oxidative stress in patients with inadequate glycemic control [64]. Furthermore, dapagliflozin has been shown to improve systemic endothelial dysfunction and arterial stiffness, independent of the blood pressure and blood glucose levels [65]. Lee et al. also reported that dapagliflozin improves vascular smooth muscle dysfunction with alterations of gut microbiota in type 2 diabetic mouse [66]. Uthman et al. demonstrated the anti-inflammatory action of SGLT2 inhibitors by showing that empagliflozin rescued the TNF-α-induced reduction of the eNOS expression in human coronary arterial endothelial cells [67].

From the perspective of inflammation, empagliflozin is suggested to promote browning of white adipose tissue (WAT) by polarizing M2 ATMs [68]. Furthermore, dapagliflozin attenuates cardiac fibrosis by promoting M2 macrophage polarization in myocardial infarction in rodents [69]. As such, the inhibition of SGLT2 may shift the macrophage polarity to an M2 status, and thus, prevent metabolic disorders causing endothelial dysfunction.

### 4.2. GLP-1 Receptor Agonists

GLP-1 receptor (GLP-1R) is expressed not only in pancreatic β-cells but also in various tissues and organs, including endothelial cells, fat, brain, heart, liver, and muscle, and both GLP-1 and GLP-1R possess pleiotropic effects [70,71]. From the standpoint of vascular protection, the usefulness of GLP-1R agonists has been reported in basic research. For example, GLP-1R agonist reduces the production of inflammatory cytokines [72,73] and apoptosis of endothelial cells [74] and induces eNOS production [75]. The PI3K/Akt-eNOS activation pathway has been suggested as an underlying mechanism [76]. Cai et al. reported that GLP-1R agonists treatment induces a protective effect on endothelial cells through a GLP-1R-ERK1/2-dependent manner [77]. Furthermore, recent trials have shown that exenatide, a commonly used GLP-1R agonist, improves the endothelial function in patients with type 2 diabetes and pre-diabetes [78,79,80].

As is the case with SGLT2 inhibitors, GLP-1R agonist is suggested to modulate macrophage polarity. The reprogramming of the macrophage phenotype towards the M2 phenotype has been shown in mice with apoE and IRS2 deficiency treated with lixenatide. This was associated with a reduction in the atheroma plaque size [81] and the regression of the early stage of atherogenesis [82]. However, these studies were only performed in mouse models. Further mechanistic investigations will be required in order to elucidate the precise role of GLP-1R agonists in macrophage polarity.

### 4.3. DPP-4 Inhibitors

Dipeptidyl peptidase-4 (DPP-4) is released from adipose tissue and acts as a pro-inflammatory adipokine, mediating local inflammation, insulin resistance, and metabolic syndrome [83,84]. The expression of adhesion molecules and inflammatory cytokines is attenuated by DPP-4 inhibitor [85]. In addition, DPP-4 inhibitors exert anti-diabetic and myocardial protective effects through the activation of PI3/Akt signaling and eNOS [86].

Of note, DPP-4 has been shown to regulate inflammation and insulin resistance in the setting of obesity by modulating the macrophage polarity. For instance, linagliptin promotes the shift of polarity toward the anti-inflammatory M2 macrophage phenotype in liver and adipose tissue, thereby improving local inflammation and insulin resistance [87].

Furthermore, clinical data indicate that DPP-4 inhibitors, including sitagliptin [88], vildagliptin [89], linagliptin [90], and saxagliptin [91], improve endothelial dysfunction. Treatment with sitagliptin for 12 weeks significantly improved the change in FMD and increased the circulating levels of CD34, a marker of endothelial progenitor cells [92]. In addition, Kajikawa showed that saxagliptin markedly increased FMD and massively decreased stromal cell-derived factor-1α (SDF-1α), a DPP4 substrate participating in the recovery of vascular injury by recruiting endothelial progenitor cells [91]. Further clinical trials and mechanistic investigations will be required in order to validate the role of DPP4-inhibitors in the pathogenesis of vascular events.

### 4.4. Biguanides

Metformin, the most common anti-diabetic agent, upregulates the blood flow in adipose tissue and skeletal muscle [93]. The metformin-induced production of eNOS and inhibition of leukocyte adhesion, vascular aging, and endothelial cell apoptosis has also been reported. The activation of AMP-activated protein kinase (AMPK) is the underlying mechanism [94,95].

Metformin also exerts an anti-inflammatory function in endothelium and adipose tissue through multiple pathways. For example, a clinical trial using long-term metformin treatment in patients with type 2 diabetes reported its efficacy in reducing levels of plasma markers (i.e., VCAM-1 and ICAM-1) independent of changes in HbA1c [96]. In addition, metformin can mediate macrophage polarization to the M2 phenotype and subsequent inhibition of the Jun N-terminal Kinase (JNK) pathway [97].

From the viewpoint of clinical trials, many prospective studies targeted at patients with type 2 diabetes have shown that metformin treatment improves the cardiovascular prognosis independently from glycemic control. In a study dealing with type 2 diabetes patients, metformin improved both the insulin resistance and acetylcholine-stimulated flow, with a strong statistical relationship between these parameters [98]. In another study, the long-term treatment of metformin improved the plasma levels of markers of the endothelial function independent of other variables, including the weight, blood glucose level, and insulin dose [96].

### 4.5. Thiazolidinediones

Thiazolidinediones (TZDs) are antidiabetic agents that bind and activate peroxisome proliferator activated receptor γ (PPARγ), which is a nuclear receptor superfamily that improves insulin sensitivity. In addition, TZDs have attracted growing interest because of their biological activities, such as their anti-inflammatory, antitumor, and anti-atherosclerotic activities [99].

PPARγ is expressed in not only adipose tissue but also endothelial cells. Endothelial PPARγ decreases the production of chemokines and adhesion molecules, such as ICAM-1 and VCAM-1, and suppresses the production of components of NOX, NOX1, NOX2, and NOX4, leading to the inhibition of generation of ROS [100]. Furthermore, PPARγ promotes NO production in endothelium and abrogates endothelin expression [101].

### 4.6. Sulfonylureas

Sulfonylureas (SUs) have been widely used for treatment of type 2 diabetes. Effects of SUs on vascular and endothelial cells is inconsistent. Studies have showed that glibenclamide, a kind of second-generation SU, have a pro-arrhythmic effect on reperfusion after an ischemic event in vivo [102] and that SUs may be coupled to an enhanced risk of congestive heart failure [103]. Meanwhile, there are reports suggesting positive effects of SUs on endothelium. For instance, it has been reported that gliclazide, one of the second-generation SUs, improves endothelial function in diabetic rabbits [104] and decreases the progression of atherosclerosis in human [105]. From the standpoint of the molecular mechanism, gliclazide has been suggested to protect endothelial cells from apoptosis by decreasing oxidative stress [106], and glimepiride also has been shown to stimulate NO production via PI3-dependent pathways in endothelium and lead to reduction of NF-κβ activation [107].

### 4.7. Medical Nutrition Therapy and Physical Activity

The aim of treatment in diabetes is to maintain an optimal level of blood glucose, lipids, and blood pressure to delay or prevent chronic diabetic complications [108]. Patients with diabetes should achieve good control of their blood glucose by following a nutritious meal plan and exercise program, losing excess weight, implementing necessary self-care behaviors, and taking oral medications or insulin therapy. Weight loss through restriction of the daily diet and physical exercise is essential for managing diabetes and preventing vascular complications. When medications are used to control diabetes, they should primarily augment lifestyle improvements.

Calorie restriction and physical exercise are known to improve not only the insulin sensitivity but also endothelial dysfunction. Calorie restriction promotes NO-dependent vasodilation and coincidentally reduces circulating ET-1 levels in patients with insulin resistance [109,110]. In addition, regular physical exercise increases the expression of vascular eNOS via PI3K/Akt-dependent phosphorylation in humans [111]. Taken together, the favorable effects of these lifestyle modifications induce increased insulin signaling, enhanced eNOS activity, and reduced inflammatory and oxidative stress, leading to the right balance between the vasodilator and vasoconstrictor actions of insulin.

Indeed, a meta-analysis Montero performed pointed out that, in patients with type 2 diabetes, physical exercise greatly increased FMD [112]. Furthermore, another recent meta-analysis revealed that aerobic and combined aerobic and resistance exercise notably improved the endothelial function in patients with type 2 diabetes, as reflected by an elevated FMD. This observation was independent of changes in cardiometabolic markers, such as the blood pressure, body mass index, and glycemic control [113].

## 5. ROCK Inhibitors as Preclinical Agents

The small GTP-binding protein Rho and its downstream Rho-associated coiled-coil containing protein kinase (Rho-kinase, ROCK) mediate a variety of cellular processes such as cell contraction, proliferation, and migration. ROCK signaling is activated by many factors, including angiotensin II, glucose, and cytokines, all of which are upregulated under diabetic condition [114,115,116]. Previous studies have elucidated ROCK as a key molecule of endothelial dysfunction. For instance, statins have been reported to inhibit the RhoA/ROCK pathway indirectly, acting by reducing the synthesis of isoprenoids. The intravenous administration of pravastatin prevented impaired NO-dependent vasodilation by blocking the activation of Rho A and Rac and the inactivation of Akt/eNOS pathways in vivo [117]. Fasudil, the first ROCK inhibitor approved for clinical use, suppresses the migration of human pulmonary microvascular endothelial cells and the proliferation of pulmonary artery smooth muscle cells caused by ET-1 [118]. Moreover, fasudil has the potential to improve endothelial dysfunction via restoring NO bioavailability in humans with atherosclerosis [119].

ROCK initiates endothelial dysfunction via NF-κB activation. IκB kinase (IKK) phosphorylates IκB through activation signals, which induces the degradation of IκBα via the ubiquitin system, leads to NF-κB RelA/p65 translocation to the nucleus, and activates the transcription of target genes. ROCK mediates the NF-κB signaling through various pathways. Our laboratory showed that ROCK regulates thrombin-mediated p65 phosphorylation and IκBα phosphorylation in endothelial cells [120]. Moreover, we reported that ROCK regulates the nuclear translocation of RelA/p65 in mesangial cells [121]. Recent researches from Antoniellis et al. showed that RhoA, an upstream factor of ROCK, controls the translocation of NF-κB (p50) in neutrophils [122]. These studies suggest that ROCK controls the nuclear translocation of multiple NF-κB components and that the way of NF-κB regulation varies depending on the stimulus and type of cells. Taken together, ROCK is a principal determinant of endothelial dysfunction.

ROCK has two isoforms: ROCK1 and ROCK2. While these sequences share 65% sequence homology, each isoform plays different roles and has unique pathways of activation. ROCK1 and ROCK2 exert different roles in endothelial dysfunction. For example, endothelium without ROCK2 has shown the reduction of chemokines and adhesion molecules through NF-κB [116]. ROCK1 is required in oxidized LDL-induced cell adhesion, while ROCK2 is involved in both endothelial adhesion and apoptosis by regulating adhesion molecules [123]. ROCK2 has been shown to be a pivotal regulator of endothelial inflammation and functions as an essential factor in the development of atherosclerosis. Because ROCK1 and ROCK2 cannot completely compensate for each other’s loss, distinctive roles of them have been pointed out [124]. ROCK2 is distributed in human vascular endothelial cells. Shimada et al. suggested that ROCK2 mediates the production of VCAM-1 and ICAM-1 and induces endothelial inflammation [125]. Furthermore, Shimokawa et al. reported that ROCK2 in a vascular smooth muscle cell (VSMC) leads to the progression of cardiovascular diseases including pulmonary arterial hypertension [126]. An elegant study from Sawada et al. suggested that loss of ROCK2 in bone marrow-derived cells decreased lipid accumulation and atherosclerotic lesions in the LDL receptor-null mice [127]. Though whether or not ROCK2 is engaged in modulating monocytic migration and adhesion toward endothelial cells is unclear, we recently demonstrated for the first time that ROCK2—but not ROCK1—is involved in the regulation of these processes [116]. These findings underscored the importance of ROCK2′s involvement in endothelial dysfunction. Therefore, ROCK2 represents an attractive target for studying critical regulators of endothelial dysfunction.

With regard to macrophage polarity, the importance of Rho/ROCK signaling has been clarified gradually. Recent studies have shown that ROCK1 and ROCK2 have different roles in the regulation of macrophage polarization into classical pro-inflammatory macrophage type 1 (M1), producing IL-12, and alternative anti-inflammatory macrophage type 2 (M2), producing TGF-β and IL-10. Though ROCK2 inhibition is suggested to result in a decreased population of M2 macrophages with the upregulation of M1 markers in age-related macular degeneration (AMD) [128], the commitment of ROCK1 and ROCK2 in the conversion of the macrophage subtype in other organs and diseases remains to be elucidated. Further mechanistic analyses will be indispensable for clarifying the role of ROCK in regulating macrophage polarity.

## 6. Conclusions and Future Perspectives

Insulin signaling pathways and endothelial cells conduct crosstalk, thus, understanding the correlation between insulin resistance and endothelial dysfunction is essential for treating diabetes-related vascular complications. Insulin resistance and endothelial dysfunction lead to the failure of NO-dependent vasodilatation, the glucose uptake by cells, and the induction of inflammation in tissues, eventually leading to atherosclerosis. In addition, endothelial NO activates VASP signaling in macrophages by increasing the M2 macrophage polarization and exerting an anti-inflammatory function under conditions of insulin resistance. A novel class of anti-hyperglycemic agents is suggested to exert their beneficial effects through this mechanism. SGLT2 inhibitors and GLP-1 agonists may promote browning of WAT by polarizing M2 macrophages and protecting against endothelial dysfunction. A firm understanding of the mechanism underlying each drug’s pleiotropic effect will be needed to establish new treatment approaches for endothelial dysfunction.
